# An experimental genetically attenuated live vaccine to prevent transmission of *Toxoplasma gondii* by cats

**DOI:** 10.1038/s41598-018-37671-8

**Published:** 2019-02-06

**Authors:** Chandra Ramakrishnan, Simone Maier, Robert A. Walker, Hubert Rehrauer, Deborah E. Joekel, Rahel R. Winiger, Walter U. Basso, Michael E. Grigg, Adrian B. Hehl, Peter Deplazes, Nicholas C. Smith

**Affiliations:** 1 Institute of Parasitology, University of Zürich, Winterthurerstrasse 266a, 8057 Zürich, Switzerland; 2 Functional Genomics Center Zürich, Winterthurerstrasse 190, 8057 Zürich, Switzerland; 3 Molecular Parasitology Section, Laboratory of Parasitic Diseases, NIAID, NIH, Bethesda, Maryland USA; 4 Research School of Biology, Australian National University, Canberra, ACT 0200 Australia; 5 School of Science and Health, Western Sydney University, Parramatta South Campus, Sydney, NSW 2116 Australia

## Abstract

Almost any warm-blooded creature can be an intermediate host for *Toxoplasma gondii*. However, sexual reproduction of *T*. *gondii* occurs only in felids, wherein fertilisation of haploid macrogametes by haploid microgametes, results in diploid zygotes, around which a protective wall develops, forming unsporulated oocysts. Unsporulated oocysts are shed in the faeces of cats and meiosis gives rise to haploid sporozoites within the oocysts. These, now infectious, sporulated oocysts contaminate the environment as a source of infection for people and their livestock. RNA-Seq analysis of cat enteric stages of *T*. *gondii* uncovered genes expressed uniquely in microgametes and macrogametes. A CRISPR/Cas9 strategy was used to create a *T*. *gondii* strain that exhibits defective fertilisation, decreased fecundity and generates oocysts that fail to produce sporozoites. Inoculation of cats with this engineered parasite strain totally prevented oocyst excretion following infection with wild-type *T*. *gondii*, demonstrating that this mutant is an attenuated, live, transmission-blocking vaccine.

## Introduction

*Toxoplasma gondii* is a zoonotic, apicomplexan parasite that belongs to the subclass Coccidia. On one hand, this protozoan is an important cause of abortion in sheep and, thereby, causes considerable economic losses^1,2^. On the other hand, the global prevalence of *T*. *gondii* infection in humans is estimated to be 30% and, in some regions, notably South America and Africa, the prevalence is much higher^3^. Although for most infected people the disease is asymptomatic, a first infection during pregnancy can lead to congenital toxoplasmosis, which may cause abortion or have serious effects for the new-born – effects that may persist for life (for example, mental retardation, hearing and loss of vision). Clinical symptoms, particularly ocular toxoplasmosis, can also occur in otherwise healthy adults but serious disease and death is more often associated with immunosuppressed individuals. Even though a small percentage of people infected with *T*. *gondii* may be affected adversely, the sheer magnitude of the number of people infected for life by this most successful and insidious of parasites means that, nevertheless, large numbers of people suffer significant morbidity as a result of infection^2–6^.

*Toxoplasma gondii* has a complex life cycle. It is able to infect a wide range of warm-blooded intermediate hosts by virtue of a highly adaptable, asexual stage, the tachyzoite, which is capable of attaching, invading, modifying and replicating rapidly within a diversity of nucleated *cells*^7,8^. Intermediate hosts mount robust immune responses^9,10^ that drive tachyzoites to convert to bradyzoites, which are persistent, asexual and infectious forms of the parasite harboured in cysts within brain and muscle cells for life. Only felids serve as definitive hosts for *T*. *gondii* and, in them, *T*. *gondii* develops through a classical coccidian life cycle^7,8^ in the epithelia of the small intestine in addition to spreading systemically as in intermediate hosts. Thus, predation of an infected intermediate host by a naïve definitive host results in bradyzoite invasion of enterocytes, initiating several rounds of rapid asexual reproduction, producing merozoites, followed by transformation into male (micro-) and female (macro-) gametes. Fertilisation of macrogametes by microgametes results in the production of zygotes, the only diploid stage in the life cycle. Resilient bi-layered walls form around the zygotes, creating oocysts. Oocysts are, initially, unsporulated and non-infectious but, given sufficient warmth, humidity and oxygen, will undergo meiosis and sporulate, ultimately producing two sets of four haploid sporozoites, contained within a second set of walled structures, called sporocysts^7,8^. These sporulated oocysts are infectious. It must be noted, however, that there is some contention about whether fertilisation is a necessary precursor to development of sporulated oocysts^11^.

At any one time, 1% of the world’s ~600 million domestic felids are shedding oocysts of *T*. *gondii* in their faeces, and an individual cat may shed up to 55 million oocysts per day for an average of 8 days; moreover, oocysts of *T*. *gondii* can survive in the environment for many months, even years^6^. In addition, cats shedding oocysts in farming areas leads to infection of livestock and development of microscopic, infectious cysts in tissues. When all these facts are combined, it is obvious that human exposure to infectious stages of *T*. *gondii*, either *via* oocysts or tissue cysts, occurs frequently. Which route of infection is more important is still debated but it is clear that, either way, every human infection results directly or indirectly from cats shedding oocysts into the environment.

Oocysts are, thus, the nexus of the life cycle of *T*. *gondii* and, therefore, interventions that interfere with the production and/or sporulation of oocysts will ultimately reduce environmental contamination and, hence, the incidence of toxoplasmosis in humans and livestock. Although we understand, quite well, the dynamics of the cell biology of sexual reproduction and oocyst wall formation in *T*. *gondii*^7,8^ and related coccidians within the genus, *Eimeria*^8,12^, we know rather less about the molecular underpinnings of these events, notwithstanding recent breakthroughs in transcriptomic^13^ and proteomic^14^ analyses of *T*. *gondii* oocysts, which have identified some potentially key structural components. We recently used a comparative RNA-Seq approach to identify genes expressed specifically in the gametocytes of *Eimeria tenella*^15^, creating a dataset that embodies a snapshot of molecules and mechanisms associated with sexual reproduction and oocyst formation. Here, we take a similar approach for *T*. *gondii* to identify candidate targets for transmission-blocking control strategies. Using data mining approaches, we have pinpointed genes coding for micro- and macrogamete-specific proteins with putatively important roles in fertilisation and oocyst wall formation. We used CRISPR/Cas9 to delete the gene for one putative fertilisation factor, HAP2, and thereby created a mutant of *T*. *gondii* that cannot form infectious sporozoites within oocysts. We demonstrated that this mutant parasite line can be used as a live, attenuated vaccine to totally prevent transmission of *T*. *gondii* by cats.

## Results

### Transcriptome sequencing of feline enteric forms of *Toxoplasma gondii*

**Figure 1:**
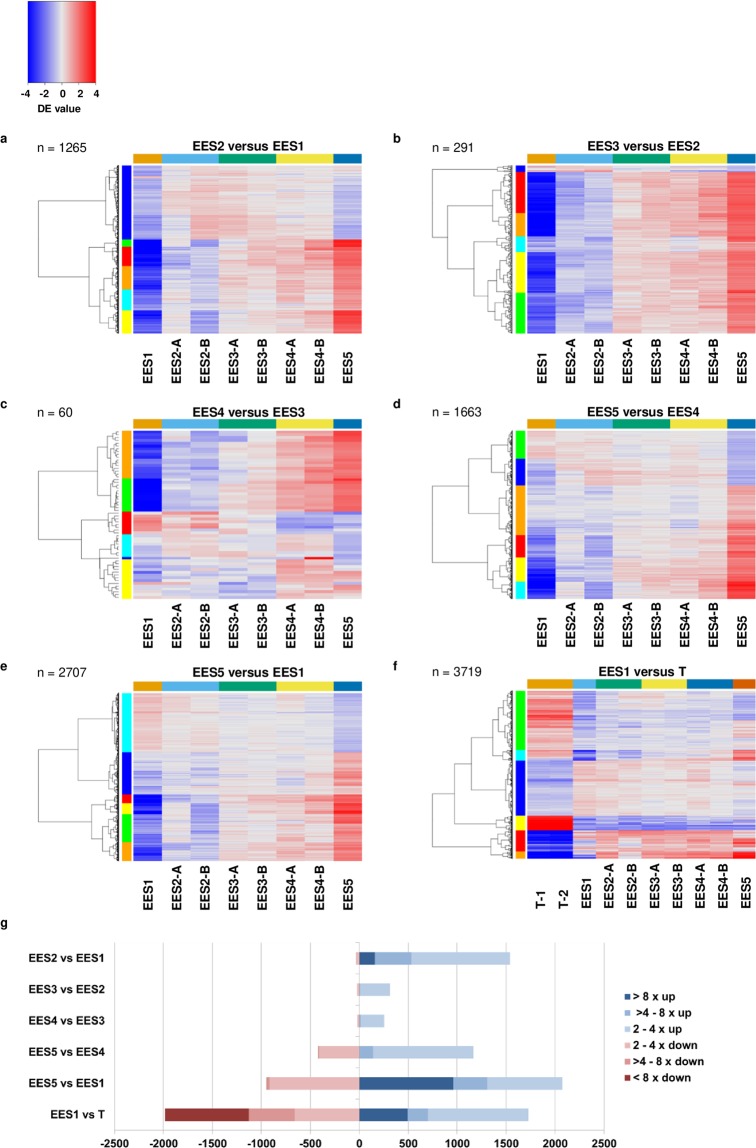
Identification of developmentally regulated transcripts as enteric development of *Toxoplasma gondii* progresses in the cat. The RNA-Seq librairies of tachyzoites (T) and different enteroepithelial stages (EES) in the *T*. *gondii* life cycle were subjected to pair-wise heirarchical clustering (EES1 = very early enteroepithelial stages isolated from one cat; EES2 = early enteroepithelial stages isolated from two cats; EES3 = mixed enteroepithelial stages isolated from two cats; EES4 = late enteroepithelial stages isolated from two cats; EES5 = very late enteroepithelial stages isolated from one cat; T = tachyzoites from two independent human foreskin fibroblast culture samples). Heat-mapping of Pearson’s correlation coefficient reveals similarities and differences in transcriptome development for (**a)** EES2 vs EES1, (**b)** EES3 vs EES2, (**c)** EES4 vs EES3, (**d)** EES5 vs EES4, (**e)** EES5 vs EES1 and (**f)** EES1 vs tachyzoites. Heatmaps show the log_2_ values of the ratio of reads between two stages. Only genes with significant differential expression (DE) are plotted; the significance threshold is set to 0.01 and the required minimum log_2_ ratio is 0.5. Coloured bars (blue, cyan, green, orange, red and yellow) on the left mark gene clusters. (**g)** Stacked bar charts show the number of differentially expressed genes with different levels of regulation in various comparisons of the EES as well as EES1 versus tachyzoites. Comparisons are performed using averages where applicable.

Samples were taken from the small intestinal epithelium of eight cats infected with *T*. *gondii* CZ clone H3 at 3, 5 and 7 days post-inoculation using our published protocol^16^. Hierarchical clustering of samples showed that time points do not necessarily reflect the progress of development (Figure S1a). We therefore used the clustering as a basis to order the samples of the enteroepithelilal stages and validated this by heat maps of genes being significantly differentially expressed (Figure S1b). Thus, samples were classified into five enteroepithelial stages (EES) of development, designated as follows: EES1 = very early (isolated from one cat); EES2 = early (isolated from two cats); EES3 = mixed (isolated from two cats); EES4 = late (isolated from two cats); EES5 = very late (isolated from one cat). Two samples of tachyzoites from independent human foreskin fibroblast (HFF) cultures were also included in analyses. Paired-end Illumina RNA-Seq reads were mapped to all 8,322 open reading frames (ORFs), representing unique proteins annotated in the *T*. *gondii* genome (www.toxodb.org) (Tables S1–S3). The variability of reads mapped to *T*. *gondii* sequences in the cat stage samples is due to the asynchronous nature of coccidian development as well as to the variability in magnitude of infection (Table S1). The highest parasite to host read ratio was achieved in late to very late enteroepithelial stages as well as in tachyzoite samples. Comparative analysis of normalised (log_2)_ read ratios between stages is depicted as heat maps (Fig. 1a–f) and scatter plots (Figure S2). The greatest stage-specific differences in mRNA levels was found between the tachyzoite and all EES samples, and between EES5 and all other EES samples. In comparison, the differences between EES1, EES2, EES3 and EES4 were relatively minor, indicating that the EES5 samples provide a useful resource for identification of genes that are differentially regulated during gametogenesis (Fig. 1g).

### Oocyst and oocyst wall-specific genes

**Figure 2:**
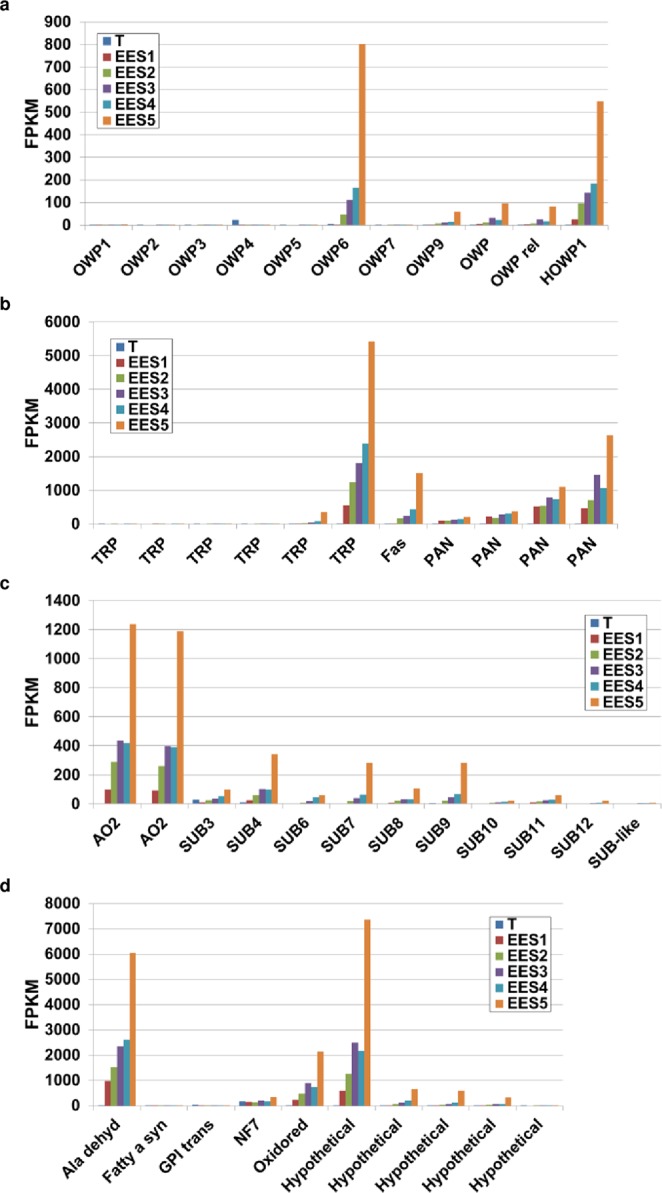
Inceased levels of transcripts coding for oocyst wall proteins, surface proteins and enzymes potentially involved in oocyst wall biogenesis. (**a**) Expression of genes coding for oocyst wall proteins (OWP) and a hypothethical OWP (HOWP1). (**b**) Genes encoding tyrosine-rich proteins (TRP), fasciclin (Fas) and PAN/Apple (PAN) domain-containing proteins. (**c**) Genes encoding enzymes involved in oocyst wall formation such as amine oxidase 2 (AO2) and subtilisins (SUB), putatively responsible for cross-linking tyrosines. (**d**) Other genes that encode proteins that have been detected in the oocyst wall^14^. GPI trans., GPI transamidase (subunit PIG); Fatty a sy., type I fatty acid synthase; Oxidored., oxidoreductase; Ala dehyd., alanine dehydrogenase; NF7, nuclear factor 7; Hypo., hypothetical protein. (FPKM = Fragments per kilobase of transcript per million mapped reads, using averages where applicable, for: EES1 = very early enteroepithelial stages isolated from one cat; EES2 = early enteroepithelial stages isolated from two cats; EES3 = mixed enteroepithelial stages isolated from two cats; EES4 = late enteroepithelial stages isolated from two cats; EES5 = very late enteroepithelial stages isolated from one cat; T = tachyzoites from two independent human foreskin fibroblast cultures).

A transmission-blocking vaccine against poultry eimeriosis has been developed; it targets the gametocyte proteins GAM56 and GAM82, which are produced in macrogametes and stored within the wall forming bodies prior to incorporation into the oocyst wall^12^. This highlights the attractiveness of oocyst wall and/or surface proteins as transmission-blocking targets. Although microarray data of sporulated and unsporulated *T*. *gondii* oocysts^13^ as well as proteomic data of oocyst versus sporocysts/sporozoite fractions^14^ are available, expression datasets for the enteroepithelial stages in the definitive cat host remain understudied. Using criteria of >4-fold increase of expression from EES5 to EES1, low expression in tachyzoites, and peak expression in EES5, we have compared mRNA levels of ORFs encoding oocyst wall proteins (OWPs) in *T*. *gondii* (Table S4) and found that read numbers mapping to *owp1*, *6*, *9*, *hypothetical owp1* (*howp1)* and two, as yet uncharacterised, *owp* orthologs (TGME49_268225 and TGME49_268230) increase from EES1 to EES5, presumably as the life cycle of the parasite progresses (Fig. 2a), supporting the idea that these are indeed genes that code for oocyst wall proteins in *T*. *gondii*. In further support of this conclusion, OWP6 has been detected previously in the oocyst wall of *T*. *gondii*^14^ and a homologue of *owp6* has also been found in the gametocyte transcriptome of *E*. *tenella*, with the corresponding protein detected in the wall forming bodies of macrogametocytes^15^.

*Toxoplasma gondii* oocyst and sporocyst walls display autofluorescence if illuminated by 405 nm laser light. This is thought to be due to the dityrosine bonds formed between tyrosine-rich proteins (TRP) in the oocyst and sporocyst wall, a model that was first proposed for *Eimeria*^17^. Accordingly, ORFs coding for *T*. *gondii* TRP were prominent in the data set of genes meeting our criteria for upregulated expression in EES5 (Fig. 2b). Additionally, expression of genes coding for enzymes proposed to be involved in the catalysis of dityrosine bond formation in the oocyst wall – AO2, an oxidoreductase and several subtilisins – was also upregulated (Fig. 2c; Table S4). Similarly, stage-specific increase in mRNA levels was observed for ORFs coding for, fasiclin and PAN/Apple domain containing proteins (Fig. 2b) as well as for the metabolic enzyme, alanine dehydrogenase, and several hypothetical proteins (Fig. 2d). These proteins/genes were identified previously in the oocyst wall proteome^14^ and/or transcriptome^13^.

### Identification of microgamete-specific genes

**Figure 3:**
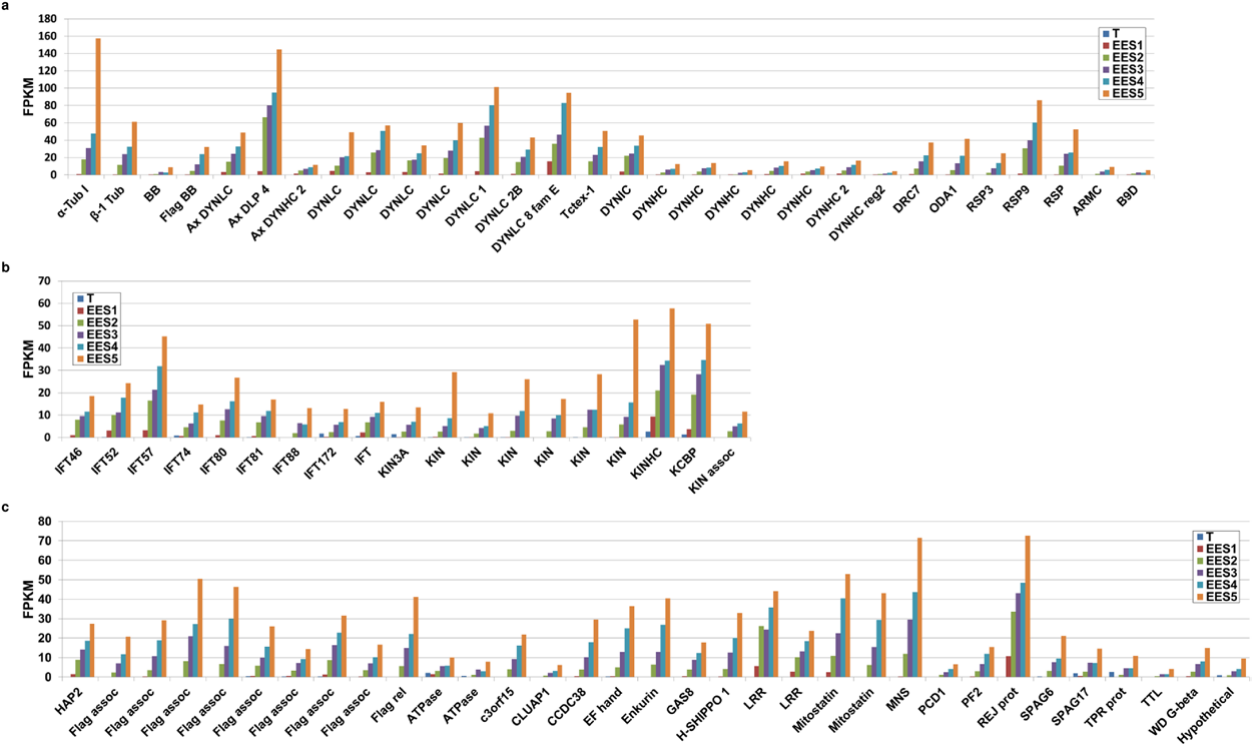
Increased levels of transcripts coding for putative microgamete proteins. (**a**) Expression of genes coding for axonemal proteins. Tub., tubulin; DYNLC, dynein light chain; Ax., axonemal; DLP, dynein light polypeptide; Tctex-1, family of dynein light chains; DYNIC, dynein intermediate chain; DYNHC, dynein heavy chain; reg., region; DNAH2, axonemal dynein heavy chain 2 family protein; ODA, outer dynein arm docking complex protein; DRC, dynein regulatory complex; RSP, radial spoke protein; BB, basal body protein; SPAG, sperm-associated antigen; ARMC, armadillo/beta-catenin family repeat-containing protein; B9D, B9 domain-containing protein. (**b)** Gene expression of flagellar transport proteins. IFT, interflagellar transport protein; KIN, kinesin; KINHC, kinesin heavy chain; KCBP, calmodulin-binding carboxy-terminal kinesin-like family protein; ass., associated. (**c)** Other microgamete genes. HAP, hapless gene; Flag assoc., flagellar-associated; Flag rel., flagellar-related; PACRG, Parkin co-regulated gene protein; MNS, meiosis-specific nuclear structural protein; PCD, primary ciliary dyskinesia protein; H-SHIPPO, human sperm tail protein; CLUAP, clusterin-associated protein; CCDC, coiled-coil protein; REJ, receptor for egg jelly; WD G-beta, tryptophan-aspartic acid G-beta repeat-containing protein; EF hand, EF hand family protein; Gas8, growth arrest-specific protein; PF2, PF2 arrest specific protein; TRR, tetratricopeptide repeat-containing protein; Mitostat., tumor suppressor mitostatin; LRR, leucine rich repeat-containing protein; Hypo., hypothetical protein. (FPKM = Fragments per kilobase of transcript per million mapped reads, using averages where applicable, for: EES1 = very early enteroepithelial stages isolated from one cat; EES2 = early enteroepithelial stages isolated from two cats; EES3 = mixed enteroepithelial stages isolated from two cats; EES4 = late enteroepithelial stages isolated from two cats; EES5 = very late enteroepithelial stages isolated from one cat; T = tachyzoites from two independent human foreskin fibroblast cultures).

**Figure 4:**
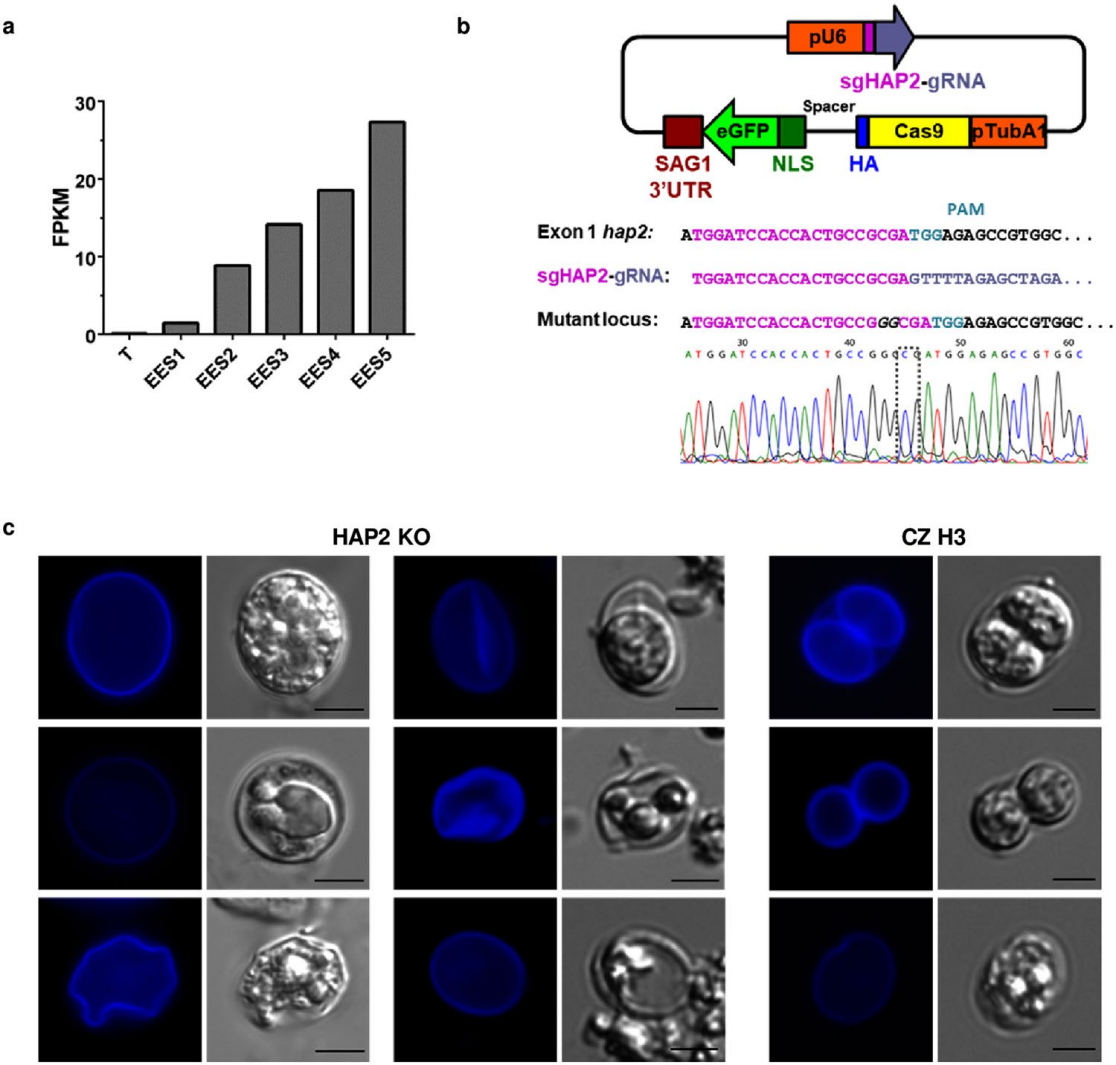
Oocysts of HAP2-deficient *Toxoplasma gondii* (HAP2 KO) exhibit aberrant morphology. **(a)** Expression of *hap2* is upregulated as development of *T*. *gondii* progresses in the cat intestine (T = tachyzoites, EES = enteroepithelial stages, FPKM = fragments per kilobase of transcript per million mapped reads). (**b)** A CRISPR/Cas9 strategy was used to delete *hap2* from the genome of *T*. *gondii* CZ clone H3. The mutant locus contains an insertion of “GG” which is shown as an alignment and in the chromatogram. (**c)** Representative oocysts and oocyst-like structures observed in CZ clone H3 and HAP2 KO parasites exploiting the autofluorescence of the oocyst/sporocyst wall under the DAPI channel and using differential interference contrast settings, demonstrating that oocysts of HAP2-deficient parasites are mis-shapen but autofluorescence is preserved, indicating formation of dityrosine bonds in the oocyst walls. Bar = 5 µm.

Our previous study^15^ identified putative microgamete-specific ORFs coding for factors involved in axoneme and flagella assembly and construction, DNA condensation, and gamete fusion in *E*. *tenella*. We therefore conducted data mining of the *T*. *gondii* RNA-Seq data using the following criteria to identify candidate microgamete-specific ORFs: >4-fold up from EES5 to EES1; low expression in tachyzoites; peak expression in EES5; annotations from ToxoDB and Blast2GO (the more comprehensive being used for descriptions) and GO terms for selection (motile cilium *etc*). Our findings are presented in Fig. 3 and Table S5. In general, we observed low FPKMs compared to other stages, which we also saw in *E*. *tenella* microgamete genes^15^. Also in keeping with our observations for *E*. *tenella*, we detected upregulation in the expression of genes coding for tubulins, dyneins, radial spokes, basal body family proteins (TGME49_283765 and TGME49_260000), a centrin family protein (TGME49_237490), kinesins, and genes coding for intraflagellar transport proteins. Upregulation of expression of the gene for SAS-6, a basal body protein known to be specific to male gametocytes in *Plasmodium*^18^ and important for fertilisation, was not detected in *T*. *gondii* EES but we did note upregulation of TGME49_297820, a gene coding for an armadillo-repeat containing protein annotated as sperm associated antigen 6 (Fig. 3a). An orthologue is abundant in the *E*. *tenella* gametocyte transcriptome^15^ and the *Plasmodium falciparum* orthologue, PF16, is part of the central apparatus of the axoneme and essential for flagellar motility and fertilisation^19^. This protein is also important for flagellar motility in *Trypanosoma brucei*^20^ and *Giardia lamblia*^21^, as well as mice^22^. Other sperm-associated genes detected included: TGME49_289260, sperm associated antigen 17: central apparatus, critical for motile cilia in mice^23^, TGME49_278710, primary ciliary dyskinesia protein 1; TGME49_207790, an enkurin thought to be involved in signal transduction in sperm^24^, which was also detected in *E*. *tenella*^15^, TGME49_293850, clusterin-associated protein 1, a key regulator of hedgehog signalling, intraflagellar transport B complex protein in mice*;*^25^ and TGME49_243550, SHIPPO-1, localised to sperm tail^26^. Finally, expression of *hap2* (TGME49_285940) was observed to increase as enteric development of *T*. *gondii* in the intestine of the cat proceeded (Fig. 3c, Table S5, Fig. 4a), in accord with our findings for *E*. *tenella*, which additionally documented specific localisation of HAP2 in microgametocytes^15^. We selected *hap2* for deletion from the *T*. *gondii* genome (Fig. 4b) to test the proof of principle that a deficiency in a key microgamete protein would (a) affect oocyst development and (b) result in a line of *T*. *gondii* that could be used to immunise cats to prevent oocyst shedding.

### Oocysts from a *Toxoplasma gondii hap2* knockout fail to sporulate

We used a CRISPR/Cas9 strategy to disrupt the *hap2* ORF in the genome of *T*. *gondii* CZ clone H3 to create a HAP2 knockout parasite (HAP2 KO) line (Fig. 4b) and examined it for off-target lesions by sequencing its genome and comparing it to CZ clone H3; in addition to the expected insertion of a “GG” mutation at the designated target site (after nt 18 of the first exon) resulting in a frameshift (Fig. 4b), we detected a second mutation in the *T*. *gondii* genome but only in an intron of TGME49_223060. Our transcriptome data indicates that TGME49_223060 is expressed consistently across all stages of *T*. *gondii* (Table S2, Figure S3a). The mutation is a deletion of “TT” at the 3’ end of intron 4 of TGME49_223060, situated upstream of a potential splicing branch point in, or upstream of, the polypyrimidine tract (Figure S3b). Experimental validation of splicing was not possible since the first five exons of TGME49_223060 are not expressed in tachyzoites. Thus, we cannot completely rule out the possibility that splicing is unaffected by the mutation but the phenotype observed in the HAP2 KO is wholly consistent with an anticipated defect in fertilisation potential (see below).

Tachyzoites of HAP2 KO *T*. *gondii* were cultured in human foreskin fibroblasts (HFFs) and used to infect mice. Brains, hearts, lungs, eyes and muscles from these mice were used to prepare infectious inocula for cats and oocysts collected from the faeces of infected cats 5–9 days later. It was only possible to recover relatively small numbers of oocysts of *T*. *gondii* HAP2 KO and no sporulation of these oocysts occurred in response to exposure to 2% H_2_SO_4_ (Table 1). Furthermore, oocysts of HAP2 KO parasites were mis-shapen though autofluorescence at 405 nm illumination was preserved (Fig. 4c), indicating formation of dityrosine bonds in the oocyst walls.

**Table 1:** Exposure to 2% H_2_SO_4_ fails to induce sporulation of HAP2-deficient *Toxoplasma gondii* (HAP2 KO) oocysts.

Days post exposure to 2% H_2_SO_4_	Percentage of CZ clone H3 oocysts sporulated	Percentage of HAP2 KO oocysts sporulated
0	0, 0, 0, 0 (0 ± 0)	0
2	46, 45, 46, 43 (45 ± 1)	0
4	69, 67, 71, 70 (69 ± 1)	0
8	79, 80, 79, 88 (82 ± 2)	0

To assess fertilisation status and ploidy of the HAP2 KO oocysts, we performed quantitative real-time PCR on DNA extracted from *T*. *gondii* CZ clone H3 and HAP2 KO oocysts. Upon fertilisation, the nuclear genome is doubled and meiosis occurs within the first few hours after fertilisation, even before sporoblast formation, to form four nuclei^27^. We amplified actin as a nuclear gene and compared its amplification by PCR with that of chaperone clp, which is encoded on an apicoplast genome fragment. The male gamete lacks an apicoplast and, therefore, oocysts contain only the maternally inherited apicoplast^28^ with a multicopy genome^29,30^ allowing the use of an apicoplast gene as a reference point. Exploiting the relative comparison method^31^, we estimated that the nuclear genome of CZ clone H3 is, as expected, 4-fold larger (range of 3.6–4.2-fold, over three experiments; Table 2) than that of HAP2 KO indicating that no fertilisation has occurred in the HAP2 KO parasites.

**Table 2:** HAP2-deficient *Toxoplasma gondii* (HAP2 KO) oocysts do not undergo meiosis.

Experiment	ΔCt_actin-clp_ CZ clone H3	ΔCt_actin-clp_ HAP2 KO	ΔΔCt CZ clone H3-HAP2 KO	n-fold change CZ clone H3:HAP KO
1	5.573 ± 0.207	7.519 ± 0.263	−1.946 ± 0.335	3.9
2	4.845 ± 0.544	6.909 ± 1.327	−2.064 ± 1.434	4.2
3	5.595 ± 0.502	7.451 ± 0.124	−1.856 ± 0.517	3.6

Quantitative real-time PCR results demonstrating the differences of Ct between actin and clp (ΔCt) ± standard deviation (SD), ΔΔCt ± SD between the CZ clone H3 and the HAP2 KO as well as an estimate of the n-fold change in the nuclear genome between the CZ clone H3 and the HAP2 KO.

### Prior infection of cats with *hap2* knockout bradyzoites prevents oocyst excretion after challenge infection with wild-type parasites

**Figure 5:**
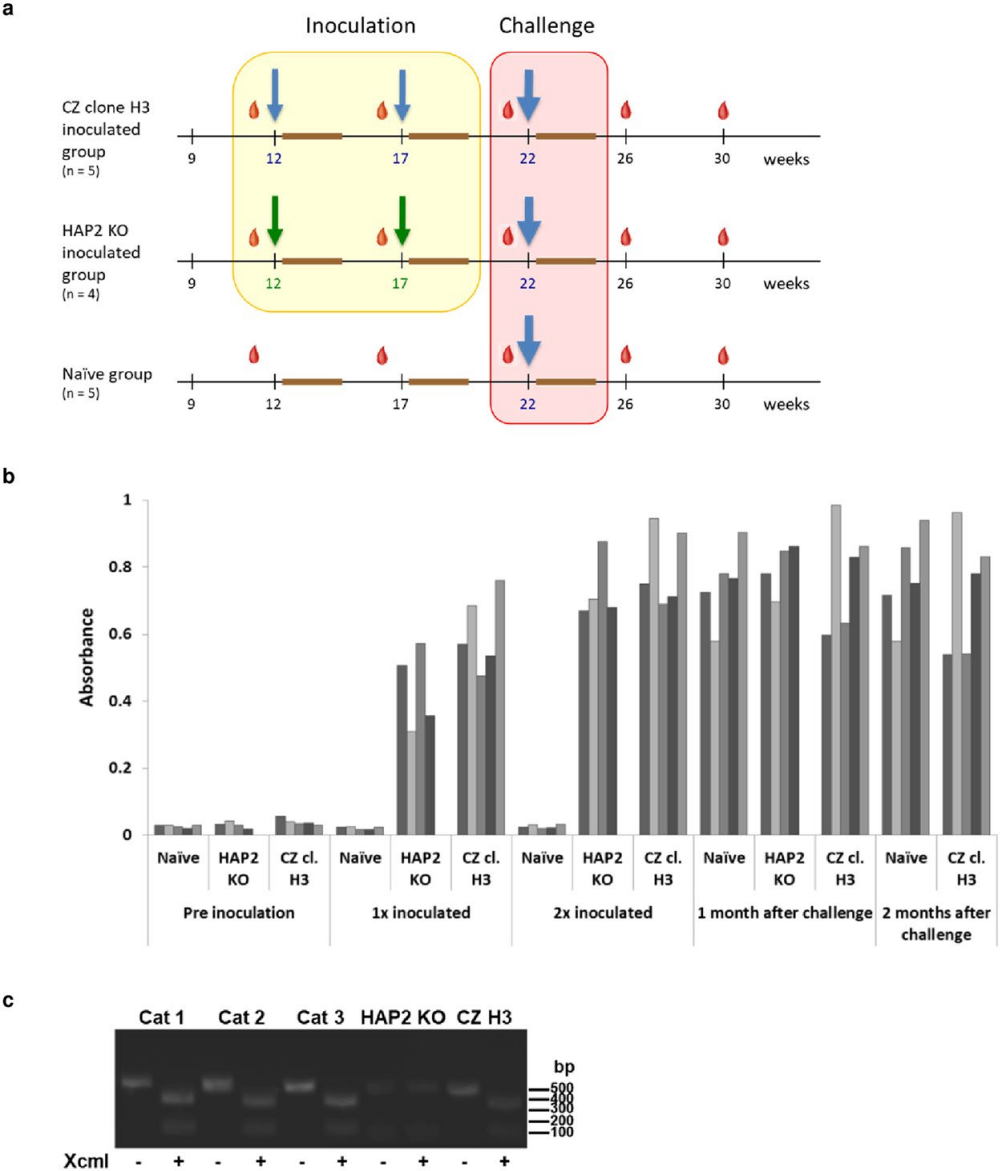
Inoculation of cats with HAP2-deficient *Toxoplasma gondii* (HAP2 KO) tissue cysts induces antibody responses but does not prevent systemic infection with wild-type *T*. *gondii* CZ clone H3 following oral challenge with tissue cysts. (**a**) Inoculation and challenge infection trial design showing age of cats, time of blood sampling of cats (red droplets), and timing of inoculations and challenge infections (arrows) on the x-axis. (**b**) ELISA results reveal formation of antibodies to *T*. *gondii*, following initial infection, by every cat. (**c**) Inoculation with HAP2 KO parasites does not prevent systemic (*ie*, tissue cyst) infection with CZ clone H3 parasites (PCR of cat brains reveals presence of CZ clone H3 DNA in cats inoculated with HAP2 KO parasites after challenge with CZ clone H3; the full length version of this image is presented as Supplementary Figure S5).

Fourteen cats were allocated into three groups of five, four and five, respectively: one group was inoculated twice, 5 weeks apart, with *T*. *gondii* CZ clone H3 bradyzoites; a second group was inoculated in the same manner with *T*. *gondii* HAP 2 KO bradyzoites; and a third group was left uninoculated (*i*.*e*., naïve). All three groups were ultimately challenged with CZ clone H3 bradyzoites, 5 weeks after the second inoculation (Fig. 5a). Blood samples were taken from all cats before the first inoculation and at 4–5 week intervals thereafter. Anti-*Toxoplasma* antibodies in these blood samples were assessed by ELISA and confirmed that, first, all cats were *Toxoplasma-*naïve prior to their first inoculation and, second, that all cats developed similar levels of anti-*Toxoplasma* antibodies following their first inoculation (Fig. 5b).

Oocyst shedding by inoculated and naïve cats was checked throughout the time-course of the challenge trial (Table 3). In the days following the initial inoculation, no oocysts were detected in the group of naïve cats. Oocysts were detected in the cats inoculated with HAP2 KO bradyzoites but only at levels below the level of reliable enumeration (*ie*, less than 1,600 oocysts in a 2day sample of faeces); moreover, these oocysts were mis-shapen, as reported above (Fig. 4c). Following the initial inoculation with CZ clone H3 bradyzoites, oocysts were first detected in faeces of cats on days 3–4 post-inoculation and oocyst shedding ceased by day 13 post-inoculation for one cat and before day 15 post-inoculation for the other four cats in this group. Peak oocyst shedding occurred on days 5–6 for all cats. Total oocyst shedding varied from 1–36 million per cat (Table 3). Such variability is not unusual amongst cats infected with *T*. *gondii*, even under strictly controlled conditions^32–34^. No oocyst shedding was observed for any cat in the days following the second inoculation. Following the final challenge inoculation with CZ clone H3 bradyzoites, none of the cats inoculated previously with either CZ clone H3 or HAP2 KO bradyzoites shed any oocysts (Table 3). However, the cats in the previously uninoculated group began shedding oocysts by day 4 (one cat) or day 5 (four cats) and continued to shed oocysts until day 11 (one cat), day 13 (three cats) or day 15 (one cat). Peak oocyst shedding occurred on days 5–6 for two cats and on days 7–8 for the other three cats in this group. Total oocyst output over the patent period varied from 2–149 million per cat (Table 3) demonstrating, in combination with antibody (Fig. 5b) responses, that the challenge infection was robust.

**Table 3:** Inoculation of cats with HAP2-deficient *Toxoplasma gondii* (HAP2 KO) tissue cysts prevents oocyst shedding on subsequent challenge with wild-type *T*. *gondii* CZ clone H3 tissue cysts.

Group	Total oocyst counts (×10^6^)	Total oocyst counts (×10^6^)	Total oocyst counts (×10^6^)
	1^st^ Inoculation	2^nd^ Inoculation	Challenge Infection
Inoculated with CZ clone H3	1, 4, 36, 7, 8 (11 ± 6)	0, 0, 0, 0, 0 (0 ± 0)	0, 0, 0, 0, 0 (0 ± 0)
Inoculated with HAP2 KO	+	0, 0, 0, 0 (0 ± 0)	0, 0, 0, 0 (0 ± 0)
Naïve	—	—	25, 36, 2, 44, 149 (51 ± 25)

In order to test if inoculation with HAP2 KO parasites also prevented systemic infection, brains from three cats inoculated with HAP2 KO tissue cysts and challenged with *T*. *gondii* CZ clone H3 were harvested and homogenised. These brain homogenates were enriched for tissue cysts by Percoll gradient fractionation and used to infect HFF *in vitro* cultures. Fractions from all three cats established infection in tissue culture and the tachyzoites were shown to be CZ clone H3 by PCR and subsequent diagnostic digestion of the PCR products (Fig. 5c; full gel presented as Figure S5); thus, whilst PCR products from tachyzoites derived from cat tissue cysts that should be a size of 475 bp could be digested with XcmI to 353 bp and 120 bp, PCR products from HAP2 KO tachyzoites gave rise to a product of 0.5 kb that could not be digested. Analysis of the nucleotide sequence of the PCR product (data not shown) confirmed the CZ clone H3 phenotype of the tachyzoites. This indicates that although prior inoculation with HAP2 KO parasites totally prevents subsequent oocyst shedding, systemic infection can still be established by the wild-type parasites used in the challenge infection.

## Discussion

In this study, we have used a comparative RNA-Seq transcriptomics approach to identify hundreds of genes putatively expressed specifically in the micro- and macrogametocytes of *T*. *gondii* (Figs 1–3, Tables S2–S5). This extensive data set provides a snapshot of the molecules that are potentially most important for the completion of the sexual phase of development of *T*. *gondii* and the formation of its oocysts, which are critical for transmission of this parasite from its definitive feline host to innumerable potential intermediate hosts. In combination with recently completed transcriptomes of *E*. *tenella*^15^ and *Cryptosporidium parvum*^35^, this transcriptome of the feline enteric stages of *T*. *gondii* provides a powerful comparative data set to facilitate identification of genes and proteins that have important and conserved functions in sexual stage biology of the Coccidia – these are all possible targets for novel, transmission-blocking control strategies, including subunit vaccination. The ability of inoculation of cats with a chemically-induced mutant of *T*. *gondii* (designated T-263^36^) to prevent oocyst excretion on subsequent challenge infection^37^ speaks to the validity of this proposal since this mutant develops normally through to micro- and macrogametocytes but fails to produce oocysts^38^, however, it must be acknowledged that, being a chemically-induced mutant line, T-263 likely harbours multiple mutations that are, as yet, undefined, apart from some enzymatic deficiencies^39,40^.

Prominent amongst the genes whose expression is upregulated in the sexual stages of *T*. *gondii* are those coding for proteins already highlighted as playing critical roles in coccidian sexual biology including tyrosine-rich and cysteine-rich oocyst wall proteins, and subtilisins and oxidoreductases thought to be involved in catalysing dityrosine cross-linking, leading to oocyst wall formation. It is now apparent that, in *Toxoplasma*, *Eimeria* and *Cryptosporidium*, the genes for oocyst wall proteins are expressed in macrogametocytes and the proteins stockpiled rather than generated *de novo* in zygotes. As we noted for *E*. *tenella*^15^, many additional stage-regulated genes, including many hypothetical genes, are also identified in *T*. *gondii* by RNA-Seq; further comparative and functional characterisation of these will allow fine dissection of coccidian sexual stage processes, including microgamete motility and fertilisation, in addition to oocyst wall biogenesis.

Amongst numerous putative microgamete-specific transcripts in our data, the one coding for a microgamete-specific gamete fusion protein, HAP2, particularly drew our attention because of its relatively well-defined localisation in microgametes, its role in membrane fusion leading to fertilisation and zygote formation in *Plasmodium*^41,42^ and *Babesia*^43,44^, and its prominence in the microgamete transcriptome of *E*. *tenella*^15^, all of which underscores its vital, conserved function in sexual reproduction in the Apicomplexa^45^. We engineered a HAP2 KO mutant of *T*. *gondii*, using a CRISPR/Cas9 strategy. We sequenced the genome of our HAP2 KO clone of *T*. *gondii* to not only confirm the disruption of *hap2* expression but also to check for off-target mutations potentially introduced by our CRISPR/Cas9 approach. We thus identified a second mutation in our HAP2 KO line. Whilst we cannot be definitively sure that this second mutation does not affect the phenotype of our HAP2 KO mutant, we believe this is unlikely because the mutation is in an intron of a hypothetical, MORN-repeat containing gene that is expressed, apart from exons 1–5, consistently across all stages of development of *T*. *gondii*, and not specifically in late enteroepithelial stages of development. In any case, this mutant parasite proved eminently suitable for answering questions about the link between fertilisation and oocyst formation.

Inoculation of cats with bradyzoites of HAP2 KO parasites induced robust antibody responses, indicating the success of the infection (Fig. 5b); however, only mis-shapen oocysts could be recovered from the faeces of the infected cats (Fig. 4c). These oocysts failed to sporulate in response to exposure to H_2_SO_4_ (Table 1), indicating that fertilisation had not occurred. Additionally, oocysts of the HAP2 KO line showed no evidence of diploidy and consequent meiosis (Table 2), providing further evidence that fertilisation did not occur in the HAP2 KO parasites. Taken together, these results indicate that fertilisation is crucial for the formation of viable, diploid zygotes, destined to develop into infectious, sporulated oocysts. This argues against the idea that the development of sporulated oocysts can occur without fertilisation^11^ but is in keeping with observations made in *Eimeria* indicating that fertilisation of macrogametes by microgametes is necessary for the production of viable, infectious oocysts since: (i) monoclonal antibodies to *E*. *tenella* microgametes can reduce oocyst formation^46^, and, (ii) reduced microgamete numbers lead to lower sporulation rates of oocysts^47^.

Our finding that our HAP2 KO parasites fail to complete fertilisation and undergo meiosis, and produce only small numbers of aberrant oocysts, establishes this mutant as a genetically-engineered, attenuated line of *T*. *gondii*, with a clear-cut phenotype. More importantly, the ability of inoculation of cats with HAP2 KO to totally prevent oocyst shedding by wild-type *T*. *gondii* (Table 3), establishes the proof-of-principle that interfering with fertilisation can completely block transmission of this parasite, a principle that can now be used to underpin the development of a transmission-blocking vaccine against *T*. *gondii*.

## Materials and Methods

### Parasites

The type II strain CZ was originally isolated from the faeces of a Siberian tiger at the Dvůr Králové Zoo (Czech Republic) in 2005 by Dr. B. Koudela and, subsequently, cloned by limiting dilution to yield clone H3, which has been maintained by passages between HFF, sheep and/or mice, at the University of Zurich ever since. Tachyzoites were grown in confluent HFF monolayers cultivated in Dulbecco’s Modified Eagle’s Medium (DMEM, Sigma-Adlrich) with high glucose supplemented with 10% heat inactivated foetal calf serum (FCS), additional 2 mM L-glutamine, 100 units/ml penicillin, 100 µg/ml streptomycin and 250 ng/ml amphotericin B. Tissue cysts for cat inoculations were obtained from brains of sheep or organs and muscles from mice and EES (sampled at day 3, 5 and 7) were harvested as described previously^16^.

### cDNA library construction and RNA-Seq

Samples of tachyzoites and EES of *T*. *gondii* for cDNA library preparation and RNA-Seq analysis were prepared as described previously^16^. RNA quality was assessed using the Agilent RNA 6000 Pico or Nano Kits (Agilent) and a Bioanalyzer 2100 (Agilent). RNA concentration was measured using a Qubit fluorometer (Invitrogen) together with the RNA assay (Invitrogen).

The TruSeq RNA Sample Prep Kit v2 (Illumina Inc.) was used in the succeeding steps. Briefly, total RNA samples (100–1000 ng) were poly-A enriched and then reverse-transcribed into double-stranded cDNA. The cDNA samples were fragmented, end-repaired and polyadenylated before ligation of TruSeq adapters containing the index for multiplexing. Fragments containing TruSeq adapters on both ends were selectively enriched by PCR. The quality and quantity of the enriched libraries were validated using a Qubit (1.0) Fluorometer and the Caliper GX LabChip GX (Caliper Life Sciences, Inc.) and qPCR. The libraries were normalised to 10 nM in Tris-Cl, pH 8.5 with 0.1% Tween-20 based on the qPCR values.

The TruSeq PE Cluster Kit v3-cBot-HS or TruSeq SR Cluster Kit v3-cBot-HS (Illumina, Inc.) was used for cluster generation using 10 pM of pooled normalised libraries on the cBOT. Sequencing were performed on the Illumina HiSeq 2500 paired end using the TruSeq SBS Kit v3-HS (Illumina, Inc.). Tachyzoites were sequenced on quarter lanes at 2 × 100 bp; EES were sequenced on 0.5 or 1 lanes each at 2 × 100 bp or 2 × 125 bp. Where few reads were obtained, the samples were sequenced twice.

Read data were quality controlled with Fastqc and FastqScreen to confirm reads were of high quality and free of contaminants. Read-alignment was done using the STAR-aligner^48^. As reference, we used the genome of *T*. *gondii* strain ME49 (ToxoDB Release 24). We computed gene expression values (normalised counts) with the function featureCounts from the R package Rsubread^49^. Developmental progress was assessed by hierarchical clustering of the samples and re-assigned to groups EES1–5 (see results, Figure S1). Differential expression was computed using the generalised linear model implemented in the Bioconductor package DESeq 2^50^ yielding the log2 value of the fold changes, p-values and false discovery rates (FDR). We considered a fold change of >4 for differential expression and used a p-value cut-off of 0.05 to determine significance. Genes with more than 10 normalised counts were considered expressed (is present = TRUE). We also calculated an additional value for transcript abundance normalised to transcript length: FPKM (fragments per kilobase of exon model per million mapped reads). The raw data and FPKMs have been deposited in NCBI’s Gene Expression Omnibus^51^ (Accession number GSE108740). Further annotation was performed by gene ontology mapping using Blast2Go^52^ v4.1.9 using an upper cut-off e-value of <0.0005.

### Construction of HAP2 knockout and sequencing

We employed a CRISPR/Cas9 strategy to insert a frameshift within the first 20 nt of the first exon of *hap2* in the CZ clone H3 *T*. *gondii*, *with* consequential disruption of the final translated HAP2 protein. First, the tubulin A1 promoter from the CZ clone H3 genome was amplified and inserted into KpnI, NsiI, digested plasmid to exchange the SAG1 promoter in the vector described previously^53^. Inverse PCR was used to exchange the sgRNA of UPRT with the sgRNA for HAP2 with Ph-sgRNA_TgHAP2mutF (5′-TGGATCCACCACTGCCGCGAGTTTTAGAGCTAGAAATAGC-3′) and Ph-genCas9mutR (5′-AACTTGACATCCCCATTTAC-3′) to yield plasmid pTub1::CAS9-U6::*sgHAP2* (Fig. 4b). Transfection of CZ clone H3 was carried out as described^53^. Twenty-four hours post-transfection, transiently transfected GFP + parasites were purified by flow cytometry as described previously^54^ (Figure S4) and the HAP2 KO clone E6 clone was further purified using two rounds of limiting dilution cloning. Sanger sequencing of PCR products using HAP2ko-dia-F (5′-GAAACAGCACTACAGCTCTTCGC-3′) and HAP2ko-dia-R (5′ ATGCATGAACAAGGTATGGTTCTGC-3′) was used to confirm the knockout. Sanger sequencing of PCR products using HAP2ko-dia-F (5′-GAAACAGCACTACAGCTCTTCGC-3′) and HAP2ko-dia-R (5′ ATGCATGAACAAGGTATGGTTCTGC-3′) was used to confirm disruption of the *hap2* ORF.

For whole genome sequencing, genomic DNA from *in vitro* cultured parasites from HAP2 KO and CZ clone H3 was extracted with the QIAamp DNA Mini Kit (QIAGEN) using the manufacturer’s protocol. DNA was sheared using Covaris Adaptive Focused Acoustics^TM^ (AFA) technology using settings specific to the fragment size of 550 bp. The fragmented DNA was size selected using AMpure beads (Beckman-Coulter), end-repaired and polyadenylated. Using the TruSeq HT DNA NanoSample Prep Kit v2 (Illumina Inc.), TruSeq adapters containing the index for multiplexing were ligated to the fragmented DNA which was then selectively enriched by PCR. The quality and quantity of the enriched libraries were validated using Qubit® (1.0) Fluorometer and the Tapestation (Agilent). The libraries were normalised to 10 nM in Tris-Cl 10 mM, pH 8.5 with 0.1% Tween-20. The Hiseq. 4000 PE Cluster Kit (Illumina) was used for cluster generation using 8 pM of pooled normalised libraries on the cBOT V2. Paired end sequencing with 2 × 150 bp was performed on the Illumina HiSeq. 4000 using the HiSeq. 3000/4000 SBS Kit (Illumina).

Using Trimmomatic^55^, raw reads were trimmed of the sequencing adapters and further trimmed 50 bases from the 3′ end to remove low-quality ends. A minimum average Phred quality of 25 and a minimum length of 25 bases were adopted. The sequences passing these filters were aligned to the genome of *T*. *gondii* ME49 (Release 33) using Bowtie2^56^ with default settings. Putative variants were then identified by following GATK best practices for DNA-sequencing^57,58^ and variants with an alternative allele frequency below 10% in the CZ clone H3 and above 90% in the HAP2 KO were marked as HAP2 KO-specific variants.

### Sporulation assay

Sporulation of oocysts of *T*. *gondii* was assessed by seeding small Erlenmeyer flasks, containing 5 ml of 2% sulphuric acid, with oocysts of the CZ clone H3 or the HAP2 KO parasites. The flasks were left at room temperature and stirred twice per day. For the CZ clone H3 oocysts, four flasks were set up and around 100 oocysts were assessed for sporulation under a light microscope from each flask on day 2, 4 and 8 after seeding. In the case of the HAP2 KO oocysts, it was not possible to set up a series of Erlenmeyer flasks because of the low number of oocysts recovered; hence, in this case, sporulation was undertaken in a Petri dish and 1000 oocysts were assessed for sporulation.

### Quantitative real-time PCR

To extract genomic DNA, oocysts were suspended in ice cold water and neat Triton X-100 was added to the suspension until foam appeared. The detergent was washed away and the oocysts were treated with 14% bleach on ice for 10 min. The bleach was then diluted with H_2_O, and oocysts were washed with ice cold H_2_O before DNA extraction or freeze drying. Genomic DNA was extracted from either freshly treated or freeze-dried oocysts using the Faecal DNA Miniprep kit from Zymo following the manufacturer’s protocol. Real-time PCR was performed using the QuantiFast SYBR Green PCR Kit (QIAGEN) on the QuantStudio 7 Flex Real-Time PCR System (Applied Biosystems) and the primers Act1-F (5′-GGCGAACCGTGAGAGAATGA-3′) with Act1-R (5′-ACAGAGAAAGAACGGCCTGG-3′) or clp-F (5′-ATTATGCGGTCCAAGCGGAA-3′) with clp-R (5′-CTACATATCCTGGAGGCGCTC-3′), resulting in amplicons of 89 bp and 158 bp, respectively. The cycling conditions used included denaturation at 95 °C for 5 min followed by 40 cycles of 10 s at 95 °C and 30 s at 60 °C. Melt curve determination was performed for 15 s at 95 °C, 1 min at 60 °C and 15 s at 95 °C. All reactions were done in triplicate. Data were analysed using the comparative Ct method^31^.

### Inoculation and challenge trial design

Fourteen 9-week old European specific pathogen free (SPF) cats were purchased from Isoquinem S.L. (Barcelona, Spain) and allowed 3 weeks to acclimatise to their new surroundings at the University of Zurich before the challenge trial. The cats were tested by ELISA (see below) for the presence of antibodies to *T*. *gondii* and for oocysts in their faeces prior to the start of the inoculation and challenge trial; all cats were seronegative and all faeces examined were oocyst-free. Each cat was vaccinated twice with Feligen CRP ad us. vet. (Virbac AG, Switzerland) prior to commencement of the challenge trial. The cats were free to move about, in three groups of four, five and five cats, in three rooms of approximately 10 m^2^, 11 m^2^ and 11 m^2^, respectively. Figure 5a outlines the design of the inoculation and challenge trial: one group of five cats was inoculated at 12 and 17 weeks of age with tissue cysts of *T*. *gondii* CZ clone H3 and challenged at 22 weeks of age with tissue cysts of *T*. *gondii* CZ clone H3; the group of four cats was inoculated at 12 and 17 weeks of age with tissue cysts of *T*. *gondii* HAP2 KO and challenged at 22 weeks of age with tissue cysts of *T*. *gondii* CZ clone H3; and the final group of five cats was left uninoculated (*i*.*e*., naïve) until challenged at 22 weeks of age with tissue cysts of *T*. *gondii* CZ clone H3. Blood samples were taken from each cat immediately prior to inoculation and challenge at 12, 17 and 22 weeks of age as well as when the cats were 26 and 30 weeks old; these samples were analysed for antibodies by ELISA (see below). Faeces were collected at 2 day intervals after inoculation and challenge and oocysts counted (see below).

### Preparation of infectious inocula

Eight to ten-week old CBA/Rj mice (Janvier, France) and Fischer 344/IcoCrl rats (Charles River, Italy) were infected by intraperitoneal injection of 200 μl PBS containing 1,000 tachyzoites of either *T*. *gondii* CZ clone H3 (mice and rats) or the HAP2 KO isolate (mice only). The use of CBA/Rj mice and Fischer 344/IcoCrl rats as hosts for *T*. *gondii* has been described previously^59,60^. At 48 or 49 days post-injection, blood samples from five randomly-chosen mice and rats were taken and tested for antibody production by ELISA (see below); all were antibody-positive. At 50 days post-injection, rodents were killed and brains, eyes, hearts and muscles collected and diced finely until homogenised. The presence of *T*. *gondii* in the inocula was confirmed by PCR, as described previously^61^. For the first inoculation of cats, brains, eyes and hearts plus 320 g of skeletal muscle from 32 *T*. *gondii* CZ clone H3 strain infected mice and brains, eyes and hearts plus 200 g of skeletal muscle from 20 *T*. *gondii* CZ HAP2 KO strain infected mice were used. For the second inoculation of cats, brains, eyes and hearts plus 300 g of skeletal muscle from 30 mice infected with *T*. *gondii* CZ strain, and brains and hearts plus 300 g muscles of 30 *T*. *gondii* CZ HAP2 KO strain infected mice were used. For the challenge infections of cats, brains, eyes and hearts plus 560 g skeletal muscle from 39 rats infected with *T*. *gondii* CZ strain were used. For each inoculation, the preparations were homogenised and divided into equal portions, which were fed to cats individually. It should be noted that, on two out of three occasions, a significant percentage (13% and 91%) of CBA/Rj mice infected with the CZ clone H3, and on one of two occasions, a significant percentage (48%) of CBA/Rj mice infected with the HAP2 KO strain of *T*. *gondii* either died unexpectedly overnight or had to be euthanised under our animal care and ethics protocols due to the exhibition of symptoms of acute toxoplasmosis (including ruffled fur, hunched posture and > 20% weight loss), even after administration of sulfadiazine^62^ and despite the fact that brain cysts could not be observed by light microscopy in infected mice. In contrast, large brain cysts were detected readily in infected Fischer 344/IcoCrl rats and only a single rat, of sixty infected with *T*. *gondii* CZ clone H3, developed mild ocular symptoms. Thus, we used tissue cysts from rats for the final challenge inocula in our trial and now prefer this host for the production of tissue cysts of *T*. *gondii*.

### Specific antibody measurement

ELISA plates (NUNC Maxisorp, Thermo Scientific, Roskilde DK) were coated with 100 µl per well using 10 µl of *T*. *gondii* tachyzoite antigen in 1 ml coating buffer (0.1 M carbonate/bicarbonate, pH 9.6) overnight at 4 °C. Production of this antigen was as described previously^63^. The plates were washed and subsequently blocked with 300 μl buffer II (PBS (pH 7.2) with 0.02% (w/v) NaN_3_, 0.05% (w/v) bovine haemoglobin (Fluka/Sigma Aldrich, Switzerland) and 0.3% (w/v) Tween-20) per well for 30 min. All further incubation steps took place in a 37 °C humid chamber for 1 h, followed by four washes with ELISA wash buffer (physiological saline containing 0.3% Tween-20). Sera were then used in a 1:200 dilution in buffer II. Alkaline phosphatase labeled goat anti-feline IgG H + L (Southern Biotech, USA) diluted 1:4000, goat anti-mouse IgG A3562 (Sigma-Aldrich, Switzerland) diluted 1:10,000 and goat anti-rat igG A8438 (Sigma-Aldrich, Switzerland) diluted 1:4000 in buffer II were used as conjugate. Antibody reactions were visualised by adding 100 µl of substrate (1 mg/ml of 4-nitrophenylphosphate (Sigma-Aldrich, Switzerland) in 0.05 M carbonate/bicarbonate buffer (pH 9.8) containing 1 mM MgCl_2_) and the absorbance was read at 405 nm in an ELISA reader (Multiscan RC, BioConcept, Allschwil, Switzerland) after 10 min of incubation at 37 °C.

### Oocyst counting

Since we could not house cats in individual cages under our animal experimentation protocols, we had to devise novel solutions to allow us to determine individual oocyst excretion rates in our infected cats. Hence, individual cats infected with *T*. *gondii* were fed different coloured plastic beads in every meal. These coloured beads proved readily detectable and allowed us to separate faeces from each cat. Faeces were collected from 2 days post-inoculation until no oocysts were observed for at least 4 days. Faeces from individual cats were pooled every 2 days, *i*.*e*., day 1 + 2 post-inoculation, day 3 + 4 post-inoculation, day 5 + 6 post-inoculation, *etc*. Faeces were soaked in 0.1% Tween-80 (Sigma-Aldrich) overnight at room temperature.

We adapted elements of three published protocols for enumerating oocysts in cat faeces^32,64,65^. In brief, faecal samples were first homogenised using a wooden palette stick and one-tenth of each was passed through a metal strainer, washed with tap water and re-suspended to a final volume of 200 ml. A 50 ml sub-sample of this suspension was centrifuged at 1000 x g for 10 min at room temperature (Allegra X-15R centrifuge, Beckman Coulter). The supernatants of each sample were discarded and the pellets re-suspended in sucrose solution (specific density = 1.2) to a final volume of 50 ml, followed by centrifugation at 1600 x g for 10 min at room temperature, without brakes, to guarantee optimal floatation. The top 10 ml of each supernatant was collected and mixed, and 1 ml was transferred into a mini-FLOTAC chamber. The mini-FLOTAC device was left undisturbed for 10 min to allow oocysts to float to the top of the counting chamber. Two different quarters were counted under a light microscope and the mean value of these two counts was multiplied by 1,600 to obtain the total number of oocysts in the original faecal sample. In cases where oocyst numbers were particularly high, the final subsample in sucrose was diluted 1:10 or 1:100 with additional sucrose solution for counting. Every sample was counted twice, by two different assessors to ensure precision. Additionally, internal protocol tests were conducted regularly, by repeated counts of samples seeded with four different known concentrations of oocysts. In cases where oocysts could not be seen in the mini-FLOTAC chambers, a 10 μl sample was drawn from the very top of the final 10 ml flotation sample, after this sample had rested undisturbed for at least 10 mins to ensure floatation of all oocysts; this sample was placed on a glass microscope slide, with coverslip, and screened under a light microscope for the presence of oocysts. This adapted protocol for oocyst enumeration proved to be efficient and reliable, with appropriate sensitivity of detection for vaccination trials.

### Tissue cyst purification and genotyping

Freshly harvested cat brains were washed several times in ice cold PBS. Approximately, 1/6^th^ of the volume was cut into small pieces and suspended in 12–15 ml ice cold PBS and syringe homogenised using 16 G, 18 G, 20 G and 22 G needles. Ice cold PBS (to a total volume of 18–23 ml) and 0.33% Tween-80 were then added to the homogenate. Each sample was divided into two and tissue cysts were fractionated on a 90%/30% Percoll gradient as described previously^40^. The interface containing the tissue cysts was harvested and washed twice with ice cold PBS at 2000 rpm for 10 min at 4 °C. The washed fraction was syringe homogenised using 18 G, 22 G, 24 G and 26 G needles and used to infect HFFs; the resulting tachyzoites were propagated *in vitro*. Genomic DNA was extracted from tachyzoites using the QIAamp DNA mini kit (QIAGEN) as per the manufacturer’s protocol. PCRs were then performed using HAP2ko-dia-F (5′-GAAACAGCACTACAGCTCTTCGC-3′) and HAP2ko-dia-R (5′- ATGCATGAACAAGGTATGGTTCTGC-3′). Control PCRs were performed with HAP2 KO and CZ clone H3 genomic DNA from tachyzoites. Diagnostic digestions of the PCR products were performed with XcmI (NEB Biolabs) and PCR products were analysed for nucleotide sequence by Sanger sequencing (Microsynth, Balgach, Switzerland). Image acquisition was performed with an imager from the Alpha Innotech Corporation using the AlphaEase FC software (version 6.0.0).

### Ethics statement

Animal experiments were performed under the direct supervision of a veterinary specialist, and according to Swiss law and guidelines on Animal Welfare and the specific regulations of the Canton of Zurich under permit numbers 130/2012 and 019/2016, as approved by the Veterinary Office and the Ethics Committee of the Canton of Zurich (Kantonales Veterinäramt Zürich, Zollstrasse 20, 8090 Zürich, Switzerland).

## Supplementary information

Supplementary Figures

Supplemental Table S1

Supplemental Table S2

Supplemental Table S3

Supplemental Table S4

Supplemental Table 5

## Data Availability

This study is based on RNA-Seq data that are publicly available and have been deposited in NCBI’s Gene Expression Omnibus with the accession number GSE108740 (https://www.ncbi.nlm.nih.gov/geo/query/acc.cgi?acc=GSE108740). Whole Genome Sequencing (WGS) raw data can be accessed in NCBI’s Sequence Read Archive (SRA) using accession number: SRP142285.
