# Supplementary material for: An experimental genetically attenuated live vaccine to prevent transmission of Toxoplasma gondii by cats
Source: Sci Rep. 2019 Feb 6;9:1474. doi: 10.1038/s41598-018-37671-8 (PMC6365665; doi:10.1038/s41598-018-37671-8)

Supplementary Figures for:

An experimental genetically attenuated live vaccine to prevent transmission of *Toxoplasma gondii* by cats

Chandra Ramakrishnan[1#], Simone Maier[1#], Robert A. Walker[1], Hubert Rehrauer[2], Deborah E. Joekel[1], Rahel R. Winiger[1], Walter U. Basso[1], Michael E. Grigg[3], Adrian B. Hehl[1*], Peter Deplazes[1*] & Nicholas C. Smith[4*]

[1]Institute of Parasitology, University of Zürich, Winterthurerstrasse 266a, 8057 Zürich, Switzerland.

[2]Functional Genomics Center Zürich, Winterthurerstrasse 190,8057 Zürich, Switzerland.

[3]Molecular Parasitology Section, Laboratory of Parasitic Diseases, NIAID, NIH, Bethesda, Maryland, USA.

[4]Research School of Biology, Australian National University, Canberra, ACT, 0200, and School of Science and Health, Western Sydney University, Parramatta South Campus, NSW, 2116, Australia.

[#]These authors contributed equally to this work

[*]Corresponding authors and joint supervisors of the research:

ABH (adrian.hehl@uzh.ch), PD (deplazesp@access.uzh.ch),

NCS (nick.smith@parasite.org.au)

Figure S1. Hierarchical clustering and heat map of transcriptomic datasets. a Hierarchical clustering of samples using all present genes shows that sampling at distinct time points (day 3, 5 and 7) does not correspond to enteroepithelial development whilst tachyzoite and tissue cyst samples cluster with each other. Sequencing depth (data not shown) was used as a threshold for processing of only high depth data (*i.e.*, exclusion of tissue cyst samples, Day 5B and D). Distances are shown in arbitrary units with scale on the left. **b** Representative heat map showing the level of regulation of significantly differentially regulated genes (n = 291, log2 ratio ≥ 0.5, p-value ≤ 0.01). The heatmap shows a comparison of mid enteroepithelial stages (EES3) and early enteroepithelial stages (EES2). Hierarchical clustering and analysis of the heat maps resulted in a new definition of samples into five enteroepithelial stages (EES): EES1 = very early enteroepithelial stages; EES2 = early enteroepithelial stages; EES3 = mixed enteroepithelial stages; EES4 = late enteroepithelial stages; EES5 = very late enteroepithelial stages. Coloured bars (blue, cyan, green, orange, red and yellow) on the left mark gene clusters

Fig. S1

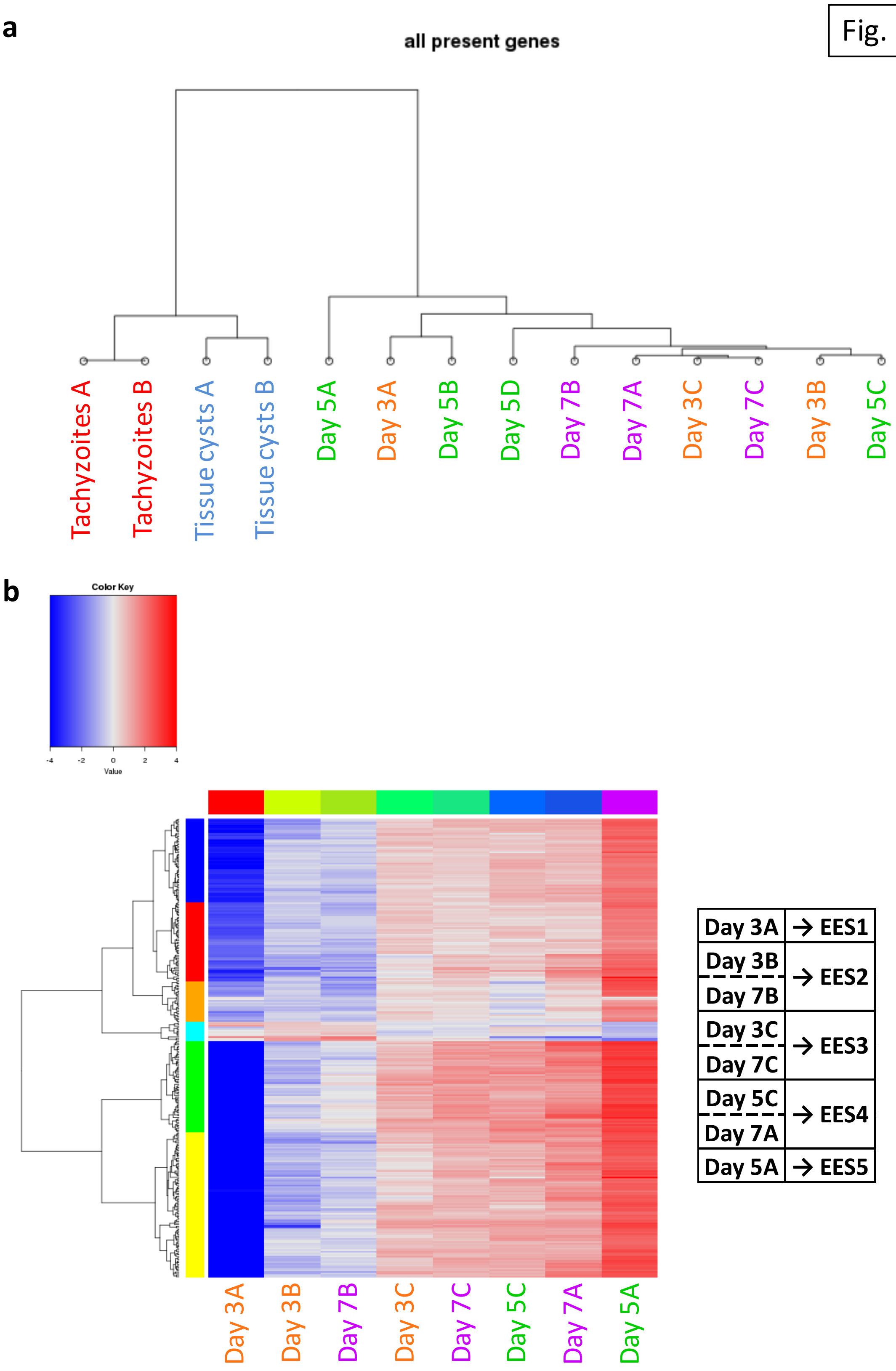
a
all present genes
Tachyzoites A
Tachyzoites B
Tissue cysts A
Tissue cysts B
Day 5A
Day 3A
Day 5B
Day 5D
Day 7B
Day 7A
Day 3C
Day 7C
Day 3B
Day 5C
b
Color Key
-4
-2
0
2
4
Value
Day 3A
Day 3B
Day 7B
Day 3C
Day 7C
Day 5C
Day 7A
Day 5A
Day 3A → EES1
Day 3B
Day 7B → EES2
Day 3C
Day 7C → EES3
Day 5C
Day 7A → EES4
Day 5A → EES5

Figure S2. Pair-wise comparison of normalised and $\log_2$-transformed gene expression values during enteric development of *Toxoplasma gondii* in the cat intestine. Scatter plots showing the expression level of all annotated, protein encoding genes. Red dots show significantly differentially expressed genes with p-value ≤ 0.01 and a $\log_2$ ratio of read numbers of ≥ 0.5. Black: No significant difference in expression. Grey: Too few reads.

Fig. S2

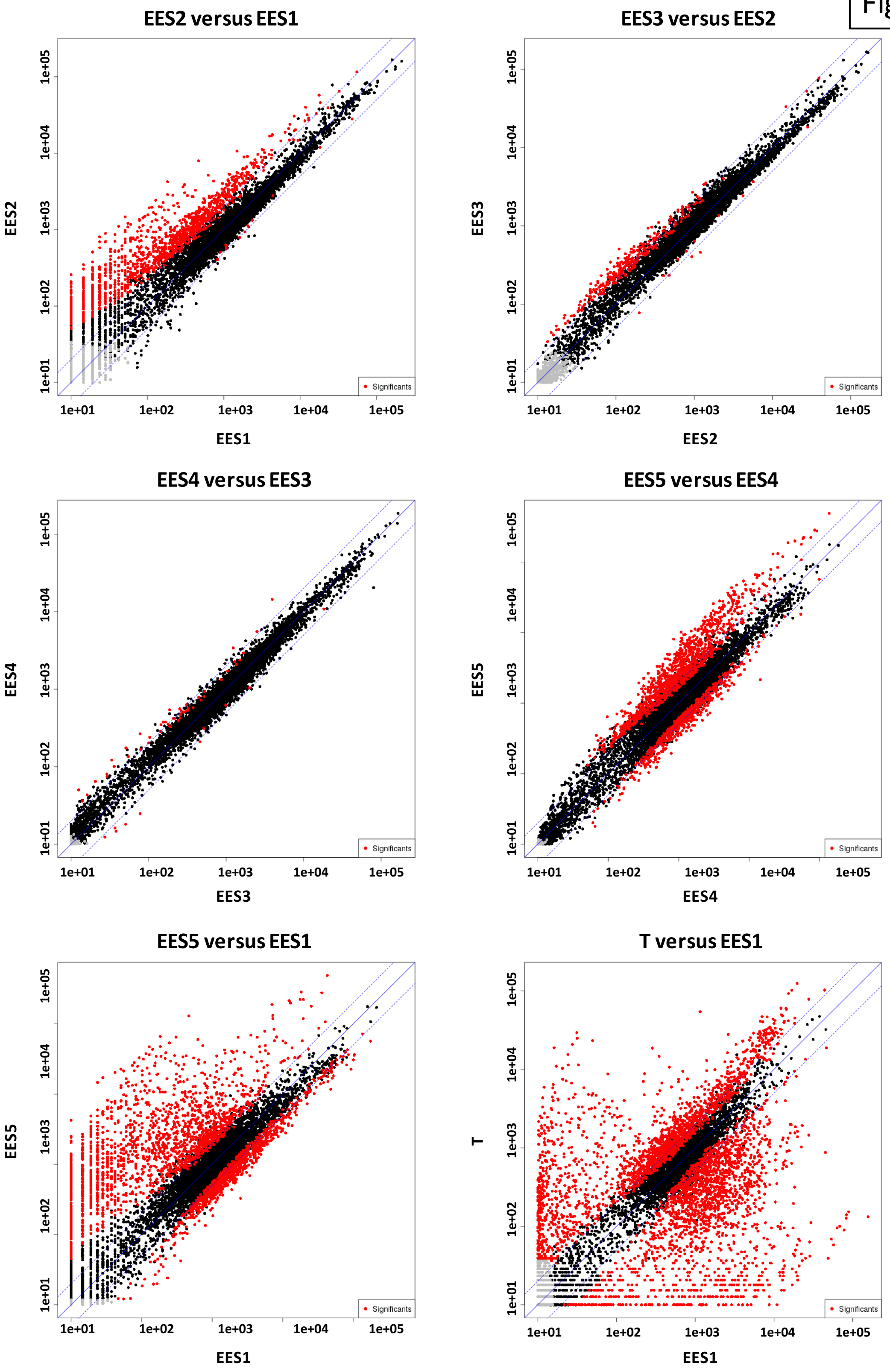

EES2 versus EES1
EES2
EES1
EES3 versus EES2
EES3
EES2
EES4 versus EES3
EES4
EES3
EES5 versus EES4
EES5
EES4
EES5 versus EES1
EES5
EES1
T versus EES1
T
EES1
1e+01
1e+02
1e+03
1e+04
1e+05
Significants

Figure S3. Expression of TGME49_223060, mutation site and gene model. a Expression of *tgme49_223060* is constant across tachyoites and feline enteric stages of *T. gondii* (T = tachyzoites, EES1-5 = enteroepithelial stages 1-5, FPKM = fragments per kilobase of transcript per million mapped reads). **b** Sequence of the 3' end of intron 4 of *tgme49_223060* in the CZ clone H3 and the HAP2 KO. Capital letters show the beginning of exon 5, letters in red highlights the splice acceptor site of intron 4. Base positions relative to exon 5 are given in blue. The double arrow highlights a potential branchpoint. **c** Gene model of, and RNA-Seq read mapping to, TGME49_223060 (from ToxoDB release 36). Read mapping shows that exons 1-5 are expressed mainly in the feline enteric stages whilst reads to almost all other exons can be found in tachyzoites, bradyzoites and, to some extent, in day 10 oocysts.

Fig. S3

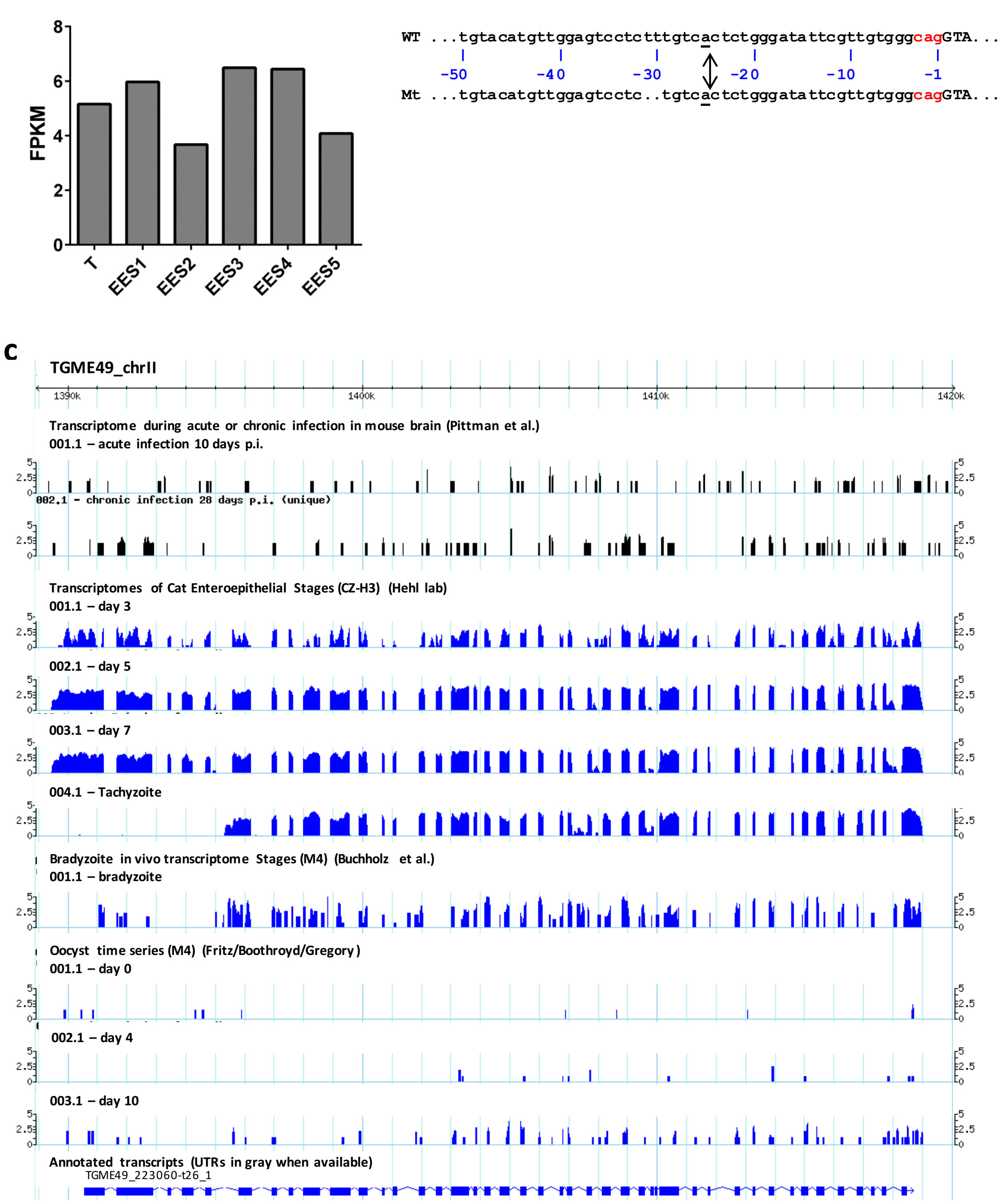
a
FPKM
0
2
4
6
8
T
EES1
EES2
EES3
EES4
EES5
b
WT ...tgtacatgttggagtcctctttgtcactctgggatattcgttgtgggcagGTA...
-50
-40
-30
-20
-10
-1
Mt ...tgtacatgttggagtcctc..tgtcactctgggatattcgttgtgggcagGTA...
c
TGME49_chrII
1390k
1400k
1410k
1420k
Transcriptome during acute or chronic infection in mouse brain (Pittman et al.)
001.1 – acute infection 10 days p.i.
002.1 - chronic infection 28 days p.i. (unique)
Transcriptomes of Cat Enteroepithelial Stages (CZ-H3) (Hehl lab)
001.1 – day 3
002.1 – day 5
003.1 – day 7
004.1 – Tachyzoite
Bradyzoite in vivo transcriptome Stages (M4) (Buchholz et al.)
001.1 – bradyzoite
Oocyst time series (M4) (Fritz/Boothroyd/Gregory)
001.1 – day 0
002.1 – day 4
003.1 – day 10
Annotated transcripts (UTRs in gray when available)
TGME49_223060-t26_1

Figure S4. Fluorescence-activated cell sorting (FACS) of transiently transfected CZ clone H3. a GFP fluorescence (x-axis) is plotted against the side scatter (SSC-A). The blue frame indicates the regions were parasites were sorted for further analysis. **b** Epi-fluorescence and phase contrast images of transiently transfected CZ clone H3 with pTub1::CAS9-U6::*sgHAP2. GFP signal can be clearly seen in the nuclei of the tachyzoite. Bar = 5 μm.*

Fig. S4

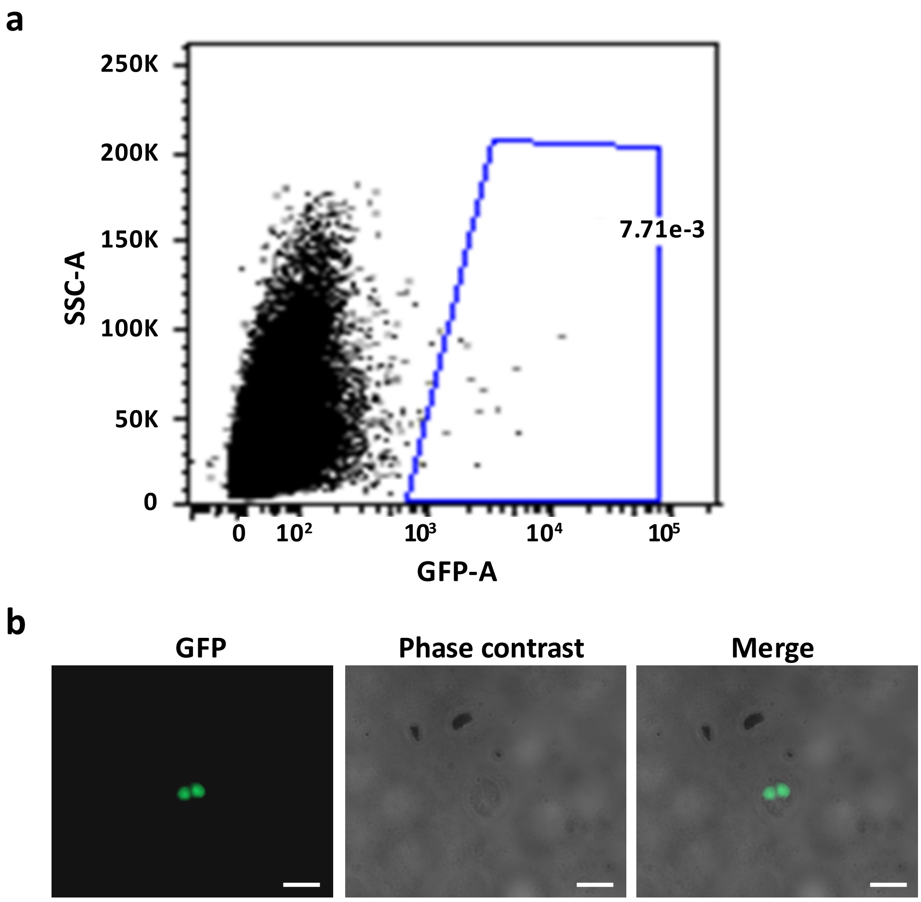

Figure S5. Inoculation with HAP2 KO parasites does not prevent systemic (*ie*, tissue cyst) infection with CZ clone H3 parasites. PCR of cat brains reveals presence of CZ clone H3 DNA in cats inoculated with HAP2 KO parasites after challenge with CZ clone H3. Image acquisition was performed with an imager from the Alpha Innotech Corporation using the AlphaEase FC software (version 6.0.0). (This is the full version of the gel presented as Figure 5d).

Fig. S5

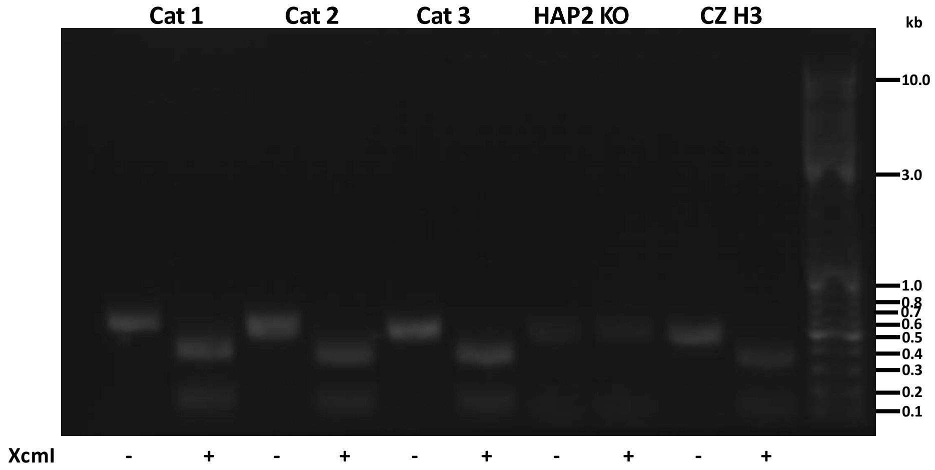

Supplement: Supplementary file 1 — Supplementary Figures [file 41598_2018_37671_MOESM1_ESM.pdf]
